# Overcoming the COVID-19 Crisis and Planning for the Future

**DOI:** 10.2967/jnumed.120.250522

**Published:** 2020-08

**Authors:** Steven H. LoGiudice, Allison Liebhaber, Heiko Schöder

**Affiliations:** 1 Department of Strategy and Innovation, Memorial Sloan Kettering Cancer Center, New York, New York; and; 2 Department of Radiology, Memorial Sloan Kettering Cancer Center, New York, New York

If you can look into the seeds of time, and say which grain will grow and which will not, speak then to me…

—William Shakespeare’s *Macbeth*

In late 2019, a new virus, severe acute respiratory syndrome coronavirus 2 (SARS-CoV-2), emerged in Wuhan, China, causing an infectious disease (1–3) that would later be named coronavirus disease 2019 (COVID-19) by the World Health Organization on February 11, 2020. This disease quickly spread across the globe, with a very rapid increase in new cases noted in many European countries and the United States between early February and early March 2020. On March 12, 2020, the World Health Organization declared the spread of COVID-19 a global pandemic. As of May 29, 2020, a total of 5,657,529 cases of COVID-19 were registered worldwide, with 356,254 confirmed deaths (4). In the United States alone, the total number of confirmed COVID-19 cases on May 28, 2020, was 1,698,523, and 100,446 Americans had died of this disease (5).

To slow the spread of disease, policy makers and public health experts in many countries have developed a catalog of measures that are applied to a variable degree in most countries worldwide, including restrictions on interpersonal contact, assembly, and travel (i.e., social distancing); recommendations for sanitation, hygiene, and the wearing of masks in public places; rapid isolation of infected patients; and use of personal protective equipment when treating affected patients. In the United States, a decline in the number of new cases and COVID-19–related deaths is now seen in New York State (6) and other areas along the East Coast. However, because SARS-CoV-2 is highly contagious, further spread of the disease is expected and appears to follow an east-to-west and centripetal pattern. Currently, no causal treatment is available for COVID-19, and a vaccine against SARS-CoV-2 is not expected until early 2021. Accordingly, researchers at the Center for Infectious Disease Research and Policy at the University of Minnesota have concluded that we could see “significant COVID activity for at least another 18–24 mo” (7). In that report, published on April 30, the authors developed 3 scenarios regarding how the COVID pandemic could unfold during this period: Scenario 1 consists of repeated peaks and valleys, scenario 2 predicts a peak in the fall of 2020 with additional smaller peaks throughout 2020 and 2021, and scenario 3 is characterized as a slow burn, with continued disease activity having smaller peaks and valleys until early 2022.

## COVID-19 AND THE U.S. ECONOMY

In early to mid-March, many U.S. state and local governments started implementing temporary restrictions on public life and nonessential work in an attempt to decrease the spread of the virus, leading to a temporary economic shutdown. Accordingly, U.S. economic output declined sharply and drastically for quarter 1 of 2020, with real gross domestic product decreasing at an annual rate of 5.0% (8). Simultaneously, the number of people filing for unemployment increased rapidly, reaching 43 million by the end of the second week in May (9). The unemployment rate increased from 3.5% in February to 14.7% in April (10), which, when considering all workers placed on temporary layoff, could have been as high as 22% (11,12). Simultaneously, U.S. retail sales declined by 16.4% (∼$80 billion) in April as compared with March 2020 (13). In early May, the U.S. government started to place increasing emphasis on the necessary economic recovery from the COVID-19–related temporary shutdown. Given uncertainties about the further spread of COVID-19, it is not surprising that predictions about the shape and speed of economic recovery in the United States vary among experts and are adjusted continuously. For instance, predictions about the decline of real gross domestic product at an annual rate for quarter 2 of 2020 are in the range of 25%–40% (14–16), with an estimated growth of −5.6% for all of 2020 as compared with 2019 (15). Of note, in the United States, consumer demand for goods and services has been a key element of economic growth over the past 50 years (17). Accordingly, as long as consumer confidence remains low (18), at least in part because of uncertainty about employment and health status, economic recovery will remain sluggish. In a recent survey among global business chief financial officers (91% from the United States), participants from all industries expressed less (!) optimism about the state of the economy and the financial prospects of their own company than during quarter 1 of 2020, and 60% did not expect a return to near-normal operating levels until sometime in 2021 (19). In another survey among global business executives, only 48% of participants from North America expected a moderate or substantial improvement in the economy (20). The Congressional Budget Office projects a robust uptick in economic growth for the second half of 2020. Nevertheless, it may take until the end of 2021 or beyond for real gross domestic product to recover to pre–COVID-19 levels (15). Finally, global supply chains could be disrupted for an extended period (21).

The *health-care economy,* of course, is not insulated from events affecting the overall economy. Since March 2020, the rapid increase in the number of COVID-19 patients requiring hospitalization and emergency treatment, as well as in public health and policy measures, has affected hospitals and private-practice groups in several ways. The first is that an onslaught of COVID-19 patients overwhelmed the capacity of some hospitals and uncovered widespread shortages in personal protective equipment.

The second is that mandated restrictions on elective surgeries and other medical procedures led to a slowdown of regular clinical operations. At the same time, non–COVID-19 patients postponed medical visits out of fear of catching the infection, leading to postponed diagnoses, procedures, and follow-up visits. Other factors for delaying care may include a widespread uncertainty about future employment, as well as financial and insurance status. According to 1 survey, by the second week of April 2020, 66% of U.S. consumers had delayed seeing their primary care physician, and 58% had delayed receiving therapies at a hospital (22). Accordingly, hospitals have been experiencing declining demand for elective procedures and surgeries as well as a decrease in the number of emergency room visits by 50% or more in the most affected areas. These trends have caused serious financial constraints on hospitals and private practices (23). The American Hospital Association recently estimated (24) a 4-mo (March–June 2020) financial impact of approximately $203 billion in losses for American hospitals and health systems. These losses are largely driven by 4 factors: the effect of COVID-19–related hospitalizations (estimated net loss, $36.6 billion), the effect of canceled or postponed medical procedures and surgeries ($164.4 billion), additional costs related to purchasing supplies and personal protective equipment ($2.4 billion), and costs due to additional financial support extended to some hospital workers for transportation and childcare ($2.2 billion).

The third way that the rapid increase in the number of COVID-19 patients has affected hospitals and private-practice groups is that a rapid rise in telemedicine has compensated only partly for the decline in expected new and previously planned medical visits. Moreover, not all patients and providers have been able to master the technologic challenges of telemedicine.

Even the strongest health systems are not immune to financial challenges driven by COVID-19. For instance, the Mayo Clinic (25) is facing a $3 billion shortfall through the end of 2020 barring interventions, and Beaumont Health’s net revenue for the first quarter of 2020 was $407.5 million less than over the same period in 2019, yielding a net income of −$278.4 million (26). Geisinger Health is projecting losses of $100 million per month. Health systems have used a series of strategies to mitigate this financial loss, including layoffs and furloughs, executive and physician pay cuts, reduced hours for hourly staff, and postponing of capital projects.

Congress has provided some financial relief as part of the Coronavirus Aid, Relief, and Economic Security Act and the Paycheck Protection Program and Healthcare Enhancement Act, allocating a total of $175 billion in relief funds to hospitals and other health-care providers. By early May, $50 billion had been distributed (27).

After the initial phase of the crisis, which now seems to have passed in some of the initially most affected areas of the United States (6) (as well as some European countries), we expect a staged and likely protracted recovery. This will require a return of patient confidence (a particular problem in the most affected urban areas) as a basis for the safe reintroduction of normal clinical operations, in which addressing chronic conditions, such as cardiovascular diseases and cancer, will again become a priority. There is considerable uncertainty about the speed of this recovery. Some previously planned procedures may still be performed with delay, but others may not: for instance, in some cancer patients, disease may have progressed, rendering them no longer suitable for potentially curative surgery; some patients with chronic diseases may have died of COVID-19; and some may have lost their employer-sponsored health insurance coverage and face financial constraints due to unemployment, further delaying their care. Some of these individuals may be eligible for Medicare or Medicaid or may seek insurance through the exchange system (28). However, reimbursement rates by these alternative insurance systems will be lower than those from traditional employer-sponsored insurance, causing further financial constraints for hospitals and medical practices. The ultimate costs from COVID-19 remain unclear. Bartsch et al. (29) have attempted to model the direct medical costs from the management of the acute infection. For a scenario in which 20% of the U.S. population get infected, they calculate direct medical costs of $163 billion, which would increase to $409 billion if 50% of the population get infected. Additional costs, such as those due to lost productivity and economic decline, and potential follow-on costs from management of chronic organ damage after acute COVID-19 infection, still would need to be added to these staggering numbers.

Against this sobering economic background, we are now going to address how health-care organizations can approach some of the COVID-19–related uncertainties and adjust their planning accordingly. To do so, they may find it helpful to create two sets of teams: one that focuses on immediate crisis mitigation (e.g., safety and infection control measures, procurement of needed supplies and personal protective equipment, and resource and staff reallocation) and another that focuses on the mid- and long-term consequences of the current crisis at all levels (e.g., patient behavior, company finances, capital planning, and expected timeline to full recovery).

## PLANNING DURING A TIME OF UNCERTAINTY

All organizations, including hospitals and health-care systems, function in the context of their external environment, and the emergence of COVID-19 can serve as a prime example of this fact. Even in normal times, uncertainty is a constant element in the development of strategy by health systems; examples include uncertainties due to unpredictable progress or setbacks in research and drug development, regulatory changes, and digital disruptions. With the increasing pace of change within the health-care industry and society, the magnitude of this uncertainty has also increased. Under normal uncertainty, planners aim to identify actions that are likely to be successful within a wide range of possible futures and ensure a high degree of agility in the execution of those actions. In other words, companies need to *identify options* that can be paused or accelerated on the basis of changes in the business environment. They also need to develop a series of *no-regrets moves* that are likely to be successful, regardless of what emerges in the marketplace (30).

The COVID-19 crisis has altered this planning process for many reasons, primarily because the degree of possible changes has expanded. This expansion results in a wider range of possible outcomes. The speed of changes has also accelerated during this crisis: whereas previously relatively little uncertainty existed over a 3- to 6-mo time horizon, the pace of change is now measured in weeks or even days.

### Modeling the External Environment

**FIGURE 1.**
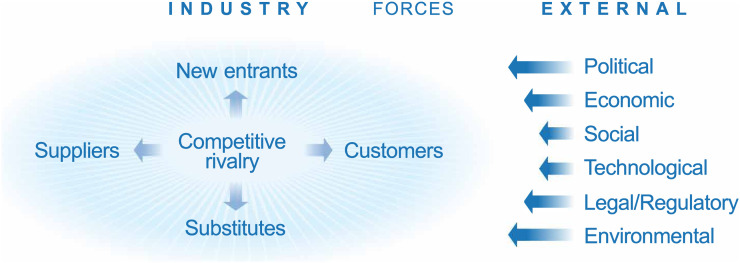
Model assessing impact of COVID-19 on health-care industry, using Porter and PESTLE frameworks.

One of the biggest current challenges for strategic planning is the unending onslaught of news and information. Placing these facts and actions into a contextual framework helps to focus attention on core dynamics each organization must understand. For this, one can use established frameworks. Here, we suggest a combination of the PESTEL framework for assessing external forces and the Porter 5-forces framework for assessing industry forces (Fig. 1). Starting with over 100 individual trends, we have used this combined framework to identify the 3 dozen most salient trends for assessing the consequences of COVID-19 on health-care systems. For each trend, we leveraged a framework created by futurist Amy Webb (31) and considered possible scenarios, ranging from pessimistic to pragmatic and optimistic. Realistically, we aimed at scenarios through the end of 2021; the range of uncertainty grows significantly over time, and forecasting beyond the 18- to 20-mo time horizon that takes us through the end of 2021 becomes unmanageable and perhaps counterproductive. Over the next 18–20 mo, we believe that social, political, economic, and legal or regulatory forces will dominate and disproportionately impact the ability of physician practices, hospitals, and health systems to recover patient volume. These forces combine in what is likely to be the most impactful trend: health-care–seeking behaviors. Modeling these forces allows an approximation of what is likely to happen; however, just like epidemiologic models (32), economic and strategic models are imperfect and require constant validation in the real world and adjustments when necessary.

### Spotlight on a Key Trend: Health-Care–Seeking Behaviors

**FIGURE 2.**
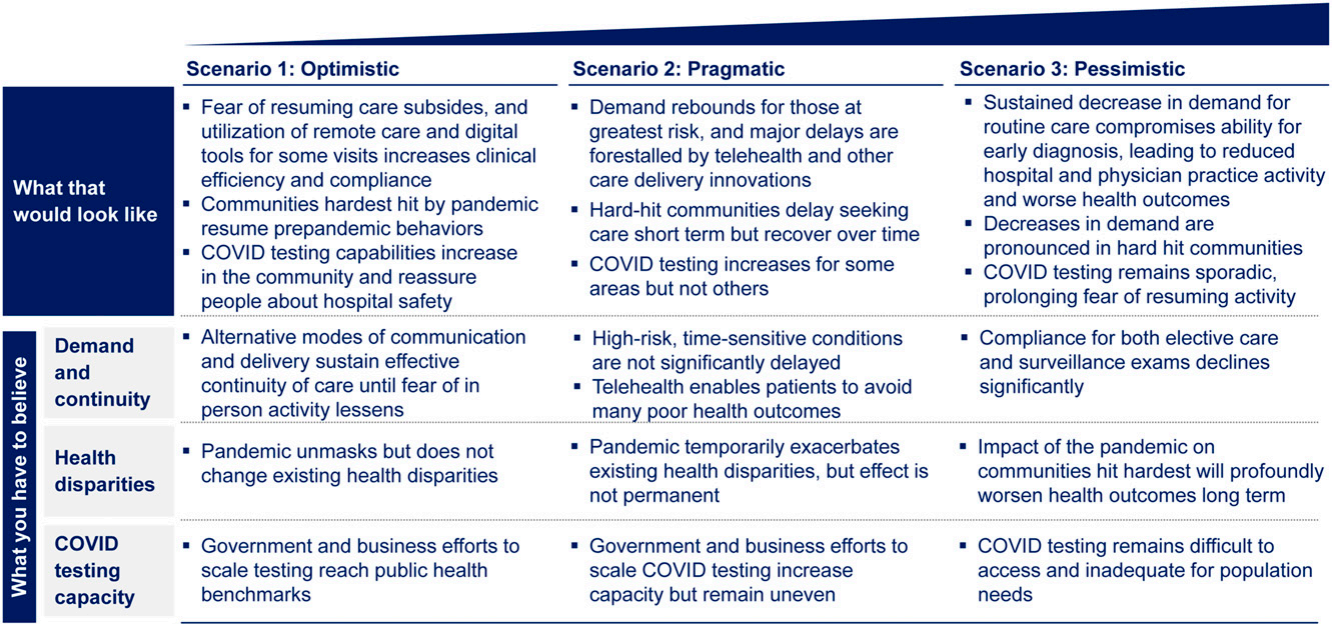
Three potential scenarios for health-care–seeking behavior.

As the pandemic emerged, the first wave of changes in clinical activity was driven by regulatory actions at the state and federal levels. By mid-March 2020, the Centers for Medicare and Medicaid Services issued its first guidance on “postponing elective surgeries, and non-essential medical, surgical, and dental procedures” (33). On March 22, 2020, New York Governor Andrew Cuomo ordered the cancellation of all elective surgical cases, a move matched by many other states. However, by mid-April, a curious dynamic had emerged across the health-care system: even for allowed procedures and acute conditions, such as myocardial infarctions (34), acute appendicitis, and acute gallbladder disease, patient volumes declined greatly. A study of Cigna claims data (35) showed that hospitalization rates for atrial fibrillation decreased 35% over the 2 mo, whereas hospitalizations for acute appendicitis and acute coronary syndromes decreased by 13% and 11%, respectively. Another study (36), by the medical record company Epic, revealed that appointments for cervical, colon, and breast cancer screenings had declined between 86% and 94% in March 2020, as compared with average volumes in the 3 prior years. Although government restrictions are now being lifted in many parts of the country (37), the demand for health-care services has not rebounded, highlighting the importance of health-care–seeking behaviors. This is also reflected by the results of a survey conducted by NRC Health in April 2020 (38), showing that 72% of respondents would not personally visit a health-care provider because of a perceived risk of getting infected. This unwillingness to physically visit a health-care facility may make it more difficult for practices and hospitals to regain volumes during 2020. Our scenario analysis framework provides a perspective on some of the likely dynamics around health-seeking behavior (Fig. 2). To predict which of these scenarios will eventually play out, we use a series of triggers and signals from inside and outside the health-care industry, including trends in ambulatory visits, trends in screening and preventive care, trends in telehealth visits versus in-person visits, transportation trends and patterns, and Google searches for *cancer*.

### Spotlight on a Key Trend: Unemployment and Insurance Coverage

**FIGURE 3.**
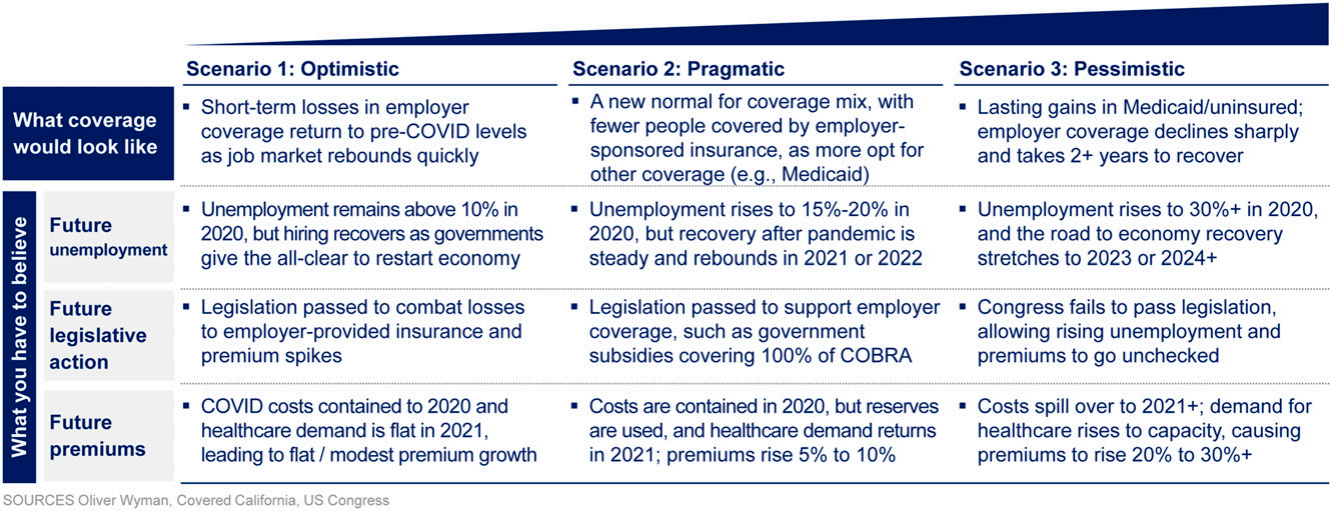
Three potential scenarios for loss of insurance coverage.

Even once fear has subsided, unemployment and insurance coverage will play an important and longer-lasting role. Individual behavior will be impacted by the loss of employer-sponsored insurance and the ability to gain insurance coverage through Medicaid, Medicare, or via an exchange. The consultancy Oliver Wyman estimates that a 15% unemployment rate will translate to an 11% decrease in the number of people covered by employer-sponsored insurance plans, or 17.1 million people (39). In this estimate, an even higher unemployment rate of 30% could translate into a 26% decrease in the number of people covered by employer-sponsored insurance plans, or 41.5 million people. Using our scenario analysis framework, we provide a perspective on these possible futures (Fig. 3), which can inform the actions an organization may take.

### Application to Hospital Planning

We are using our trend monitoring and scenarios to inform decision making and planning and to forecast possible futures for patient activity recovery. To do so, we need to translate these various forces into metrics that *quantify* the impact of changes in health insurance coverage and health-care–seeking behaviors, as well as overall economic health and pent-up demand for medical procedures. We then enter these metrics into a quantitative model. The quantification of unemployment and its impact on insurance coverage is relatively easy. However, for other factors, we must rely on proxy metrics—ideally, those that are reported regularly and are also forecasted. For instance, the Consumer Confidence Index can serve as a proxy for general economic well-being, but its utility is limited because it is not forecasted. In contrast, gross domestic product growth, a summation of overall economic activity, is also forecasted over the next 6–12 mo.

On the basis of such modeling work, we agree with the belief that recovery in the health-care sector will resemble the famous Nike swoosh (40), with slow and protracted improvement after the sharp and sudden drop that occurred in March and April. The return of confidence in the safety of hospitals will take time and will mean a slow return to normal levels of health-care activity, especially for those activities that patients feel can be delayed, such as routine annual examinations and screening mammograms and colonoscopies.

For our planning process, we start with a baseline strategy assessment (41), reviewing the organization’s last position just before the crisis and determining if these precrisis strategic actions still make sense now. We suggest placing the prepandemic strategic big bets into 3 categories: “even more important” (the urgency or need to make these bets and successfully execute on them has increased), “about right” (the bets still seem correct), and “unsure” (the importance and value of these bets is unclear in the wake of the pandemic). To help make these assessments, it is valuable to connect each of these bets with the most relevant external forces and trends so that scenario analysis can directly influence the decision making. A final consideration are *no-regrets* moves, that is, actions that are considered important for the long-term sustainability and vibrancy of the organization, regardless of the speed and shape of the recovery.

## SUMMARY AND OUTLOOK

**FIGURE 4.**
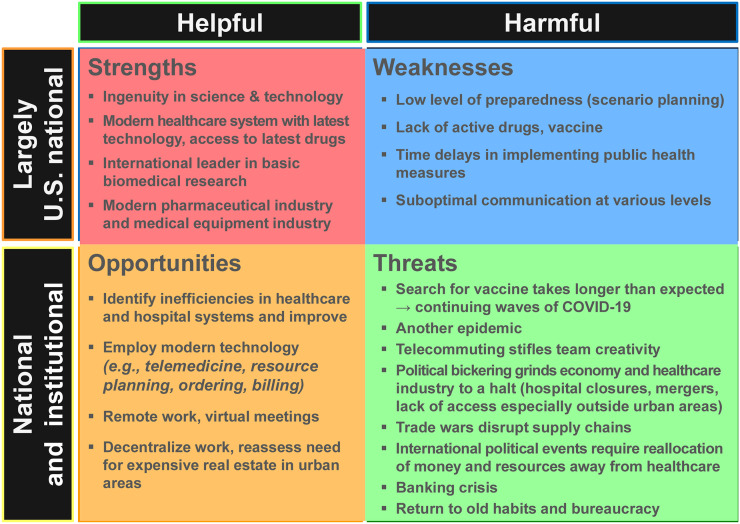
Modified analysis of SWOT for overall and health-care economic recovery in United States. *SWOT = strengths, weaknesses, opportunities, and threats.

The COVID-19 crisis came as a surprise to the United States and its health-care system. Undoubtedly, events over the past 5 mo will be investigated in greater detail in the future. However, the major aim of this article is to look forward. Figure 4 shows some potential future threats if the crisis is not mitigated quickly but also some reasons for optimism and opportunities to apply lessons learned in recent months. For instance, the crisis may unlock ingenuity in drug design and vaccine development. In the clinical arena, it has already forced the rapid introduction of telemedicine at all levels of the health-care system. If implemented properly, the latter clearly can translate into considerable time savings for patients and physicians. It will be important to maintain this spirit of innovation throughout the U.S. health-care system.

Institutions and their leaders should learn from their successes and failures in handling the primary medical and secondary economic COVID-19 crisis. A stable and sustainable path to financial recovery will require a fine balance between improving revenues and decreasing expenses. For hospitals, the main source of revenue is income from clinical operations, and the largest source of expenses is staff compensation. Faced with a decline in revenue from elective procedures, surgeries, and therapies, virtually all organizations have implemented cost-cutting measures, including, in some cases, painful cuts to salaries and benefits, and (possibly affecting the medical equipment industry) virtually systemic freezes on capital expenditures. Nevertheless, whereas cost-cutting measures may be necessary in the short term to address immediate financial imbalances, it is generally true that companies cannot save their way out of a crisis. Accordingly, the main emphasis must be on the recovery of revenue from clinical services, which in the first instance requires reestablishing patient confidence. To emerge successfully, it is just as important to retain current patients and expand the future patient pool (e.g., by expanding geographic reach and introducing new services and procedures) as to retain the talent that can offer these services. Institutions may also critically review their supply chains, the efficiency of their revenue cycle, and potential reductions in their physical footprint to cut real estate expenses. Most importantly, however, institutions *must* look beyond the day-to-day operations and develop and implement long-term initiatives and programs that will help them to defend and expand their position in the marketplace. This may be the right time to remember that “*Your best way to predict the future is to create it*” (Abraham Lincoln).

## DISCLOSURE

No potential conflict of interest relevant to this article was reported.
